# 

**DOI:** 10.1192/bjb.2024.104

**Published:** 2025-10

**Authors:** Tom Harrison

**Affiliations:** Professor of Education and Deputy Pro Vice Chancellor: Honorary Researcher in the History of Medicine Unit, Applied Health Sciences, University of Birmingham, Birmingham, UK. Email: tomharrisonjw@gmail.com

**Figure:**
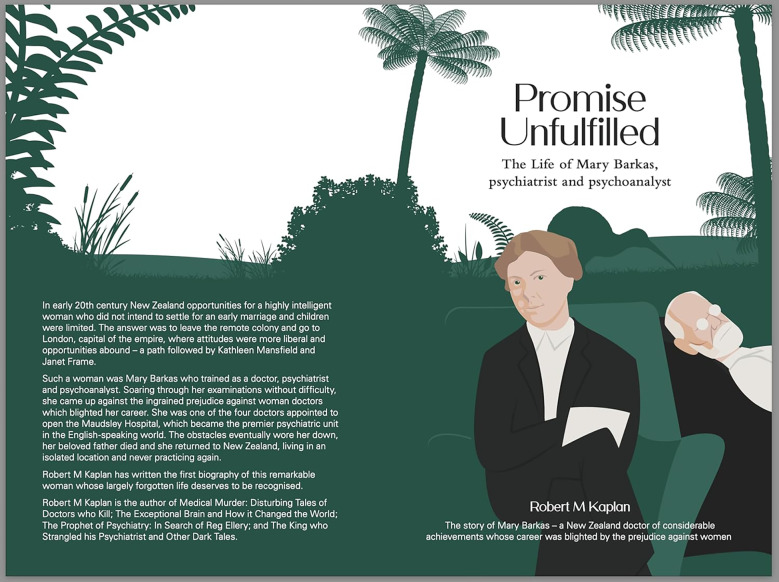

The history of women in the medical profession is dominated by the successful minority. Dr Mary Barkas in the early 20th century, despite her intelligence and commitment to her profession, succumbed to the male prejudice that prevented her achieving her full potential. That she had the capability to play a significant role in the profession is illustrated by the fact that in a period of 3 years as a founding member of the Maudsley Hospital in London she translated a number of psychoanalytic works and published a series of papers on subjects as disparate as the psychoanalytic treatment of psychosis and the neuropathology of tabes dorsalis. Previously she had qualified as one of the first female psychoanalysts, undergoing analysis with Otto Rank. Despite her evident ability and capacity for hard work, her appointments to any psychiatric role were usually of a ‘temporary’ nature, specifically because of her gender. Her letters to her father describing life in the early years of the Maudsley are themselves worthy of attention to historians of that service. Unable to gain a substantive permanent appointment, partly owing to her commitment to psychoanalysis, she resigned. Subsequently, after abandoning a particularly arduous 5-year appointment as superintendent to the failing Lawn Hospital in Lincoln, she spent the final 30 years of life in New Zealand, withdrawing any form of practice.

Her 1925 paper ‘The treatment of psychotic patients in institutions in the light of psychoanalysis’^1^ is of particular interest in that she addresses one of Freud's dictums that the treatment of psychosis is difficult because such patients are unable to establish a transference relationship. From her previous experience in mental hospitals, it was evident to her that this was patently untrue and that staff working with such people intuitively work with it. She extrapolates from this that the nurses and attendants had an active role in their recovery, rather than mere containment.

Dr Robert Kaplan has done us a great service in bringing Mary Barkas's history to light. He acknowledges that his book is but a ‘first step’ in understanding her and it would certainly be interesting to have the perspective of a social historian, perhaps bringing a female perspective. Her story is perhaps more enlightening in understanding the nature and impact of male prejudice than ones that celebrate those who win through, especially in the light of the numerous letters she wrote to her father describing her experiences.
